# Supplementary material for: Molecular Typing and Antimicrobial Susceptibility Profiles of Streptococcus uberis Isolated from Sheep Milk
Source: Pathogens. 2021 Nov 16;10(11):1489. doi: 10.3390/pathogens10111489 (PMC8624425; doi:10.3390/pathogens10111489)
Supplement: Supplementary file 1 [file pathogens-10-01489-s001.zip › pathogens-1441473-supplementary.pdf]

Supplementary Table S1.

Streptococcus uberis isolates from ovine milk included in this study. Isolates were grouped by ST, corresponding to the combination of seven genes (*arc*C, *ddl*, *gki*, *rec*P, *tdk*, *tpi*, and *yqi*L). Clonal complexes (CC) are also reported for some isolates. The new alleles and ST are indicated with an asterisk.

N Isolate	**ST**	***arc*C**	***ddl***	***gki***	***rec*P**	***tdk***	***tpi***	***yqi*L**	**CC**
n = 1	294	10	1	5	2	45	4	3	143
n = 1	350	3	2	3	3	3	2	3	86
n = 1	384	4	1	5	2	17	4	10	143
n = 1	386	1	2	3	2	1	1	35	
n = 3	562	5	1	4	2	4	4	3	143
n = 4	808	3	1	3	2	3	4	3	143
n = 1	868	3	1	3	2	3	2	3	
n = 1	910	3	58	41	2	3	4	3	
n = 1	1112	16	1	4	2	33	4	3	143
n = 2	1265	3	41	4	2	3	1	10	
n = 1	1266	4	1	11	2	46	33	30	
n = 1	1267	4	1	11	2	46	33	3	
n = 1	1268	3	2	5	3	9	2	3	
n = 1	1167*	4	1	34	2	29	4	15	
n = 1	1168*	10	1	5	1	46	4	15	
n = 1	1169*	17	7	34	2	29	4	15	
n = 1	1170*	3	2	3	2	3	4	9	
n = 1	1171*	4	2	34	2	51	4	30	
n = 1	1172*	10	2	34	1	47	33	15	
n = 2	1173*	25	1	4	3	17	1	10	
n=3	1174*	9	1	4	3	2	1	10	
n = 2	1175*	3	2	5	3	3	2	3	86
n = 3	1176*	4	1	5	2	44	4	5	
n = 4	1177*	4	1	5	2	44	4	10	
n = 1	1178*	10	1	5	2	45	4	5	
n = 1	1179*	9	1	4	3	17	1	10	
n = 1	1180*	2	2	29	2	29	4	15	
n = 1	1181*	3	1	3	4	3	4	3	
n = 3	1182*	4	42	11	2	46	33	30	
n = 1	1183*	42	2	32	2	47	33	38	
n = 1	1184*	4	1	5	2	44	4	15	
n = 1	1185*	3	2	5	3	34	4	3	86
n = 2	1186*	4	1	5	2	44	4	38	
n = 1	1187*	4	1	9	3	42	4	5	
n = 3	1188*	4	2	5	4	9	1	3	
n = 2	1189*	10	1	5	2	46	4	3	143
n = 1	1190*	3	58	41	2	3	2	3	
**N Isolate**	**ST**	***arc*C**	***ddl***	***gki***	***rec*P**	***tdk***	***tpi***	***yqi*L**	**CC**

n = 1	1191*	35	24	3	2	3	1	3	
n = 4	1192*	4	1	5	2	44	4	3	143
n = 1	1193*	9	1	4	3	44	1	10	
n = 1	1194*	10	2	4	2	47	33	38	
n = 1	1195*	5	1	4	2	33	4	3	143
n = 2	1196*	3	1	4	2	3	2	3	
n = 1	1197*	1	61	4	1	2	1	36	5
n = 1	1198*	3	1	3	1	9	4	3	
n = 1	1199*	3	1	5	2	3	2	3	
n = 1	1200*	3	2	3	2	31	4	3	86
n = 1	1231*	81*	68*	4	36*	46	43*	15	
n = 2	1232*	3	65*	3	4	5	2	3	
n = 1	1233*	10	1	5	1	46	4	75*	
n = 1	1234*	84*	10	66*	2	111*	45*	76*	
n = 1	1235*	10	67*	4	2	46	33	10	
n = 2	1236*	4	4	3	1	44	4	77*	
n = 2	1237*	10	1	5	2	45	43*	30	
n = 1	1238*	7	1	67*	2	73	4	5	
n = 1	1239*	10	67*	5	2	45	4	10	
n = 2	1240*	10	68*	5	2	45	43*	30	
n = 1	1241*	40	1	68*	2	17	4	78*	
n = 3	1242*	80*	1	4	1	13	1	3	
n = 1	1243*	5	1	70	1	13	1	10	
n = 2	1244*	3	2	3	2	110*	2	3	
n = 1	1245*	21	4	63	2	10	43*	15	
n = 1	1246*	81*	10	3	2	112*	7	38	
n = 4	1247*	42	10	70*	2	111	4	27	
n = 1	1248*	21	2	63	2	6	43*	15	
n = 1	1249*	4	1	16	1	13	4	81*	
n = 1	1250*	82*	1	5	2	45	4	38	
n = 2	1251*	42	67*	4	2	46	33	30	
n = 1	1252*	3	1	5	2	112*	7	5	
n = 1	1253*	10	67*	4	1	46	4	33	
n = 1	1254*	42	10	70*	2	29	4	27	
n = 2	1255*	4	67*	32	2	46	4	38	
n = 2	1256*	81*	10	3	2	115*	4	15	
n = 1	1257*	3	1	5	2	112*	7	62	
n = 1	1258*	83*	10	70*	2	111*	4	27	
n = 1	1259*	10	68*	6	2	45	43*	30	
n = 1	1260*	4	68*	69*	2	116*	43*	30	
n = 1	1261*	9	1	4	3	113*	1	10	
n = 1	1262*	10	67*	4	1	46	4	79*	
n = 1	1263*	2	1	4	2	114*	4	34	
**N Isolate**	**ST**	***arc*C**	***ddl***	***gki***	***rec*P**	***tdk***	***tpi***	***yqi*L**	**CC**

n = 1	1264*	40	10	69*	36*	115*	44*	80*
n = 1	1284*	71	1	3	1	54	2	3
n = 1	1285*	81*	30	30	1	115*	47*	38
n = 1	1286*	85*	66*	5	37*	109*	7	5
n = 1	1287*	81*	68*	4	36*	118*	43*	15
n = 1	1288*	2	2	69*	2	119*	4	10

Supplementary Table S2. Distribution of Sequence Types (STs) within the three defined Clonal Complex (CC)

N Isolate	ST	CC
1089	294	143
1107	384	143
1886	562	143
2210	562	143
2362	562	143
2457	808	143
2679	808	143
2717	808	143
2743	808	143
2864	1112	143
2900	1189	143
3021	1189	143
3024	1192	143
3027	1192	143
3046	1192	143
3152	1192	143
3213	1195	143
3257	350	86
3295	1175	86
3303	1175	86
3308	1185	86
3383	1200	86
3464	1197	5

Supplementary Table S3. Primer sequences for resistance genes and PCR conditions.

**Target Gene**	**Nucleotide Sequence (5'-3')**	**Annealing Temperature (°C)**	**Amplicon Size (bp)**	**Reference**
aad-6	AGAAGATGTAATAATATAG<br>CTGTAATCACTGTTCCCGCCT	37	978	[31]
*aph*A-3′	GGGGTACCTTTAAATACTGTAG<br>TCTGGATCCTAAAACAATTCATCC	50	848	[32]
*bla*Z	AAGAGATTTGCCTATGCTTC<br>GCTTGACCACTTTTATCAGC	45	517	[33]
*erm*A	AAGCGGTAAACCCCTCTGAG<br>TCAAAGCCTGTCGGAATTGG	58	440	[34]
*erm*B	CATTTAACGACGAAACTGGC<br>GGAACATCTGTGGTATGGCG	58	424	[34]
*erm*C	ATCTTTGAAATCGGCTCAGG<br>CAAACCCGTATTCCACGATT	58	294	[34]
*erm*TR	ATAGAAATTGGGTCAGGAAAAGG<br>CCCTGTTTACCCATTTATAAACG	48	376	[35]
*mef*A	AGTATCATTAATCACTAGTGC<br>TTCTTCTGGTACTAAAAGTGG	45	500	[35]
*tet*O	AACTTAGGCATTCTGGCTCAC<br>TCCCACTGTTCCATATCGTCA	50	515	[36]

*tet*L	CATTTGGTCTTATTGGATCG<br>ATTACACTTCCGATTTCGG	54	456	[37]
*tet*M	GTTAAATAGTGTTCTTGGAG<br>CTAAGATATGGCTCTAACAA	52	576	[37]
tetK	GTAGCGACAATAGGTAATAGT<br>GTAGTGACAATAAACCTCCTA	55	360	[36]
*tet*S	CATAGACAAGCCGTTGACC<br>ATGTTTTTGGAACGCCAGAG	48	667	[38]
